# *LINC01432* binds to CELF2 in newly diagnosed multiple myeloma promoting short progression-free survival to standard therapy

**DOI:** 10.21203/rs.3.rs-4888379/v1

**Published:** 2024-10-15

**Authors:** Richa Mishra, Prasanth Thunuguntla, Alani Perkin, Dhanusha Duraiyan, Katelyn Bagwill, Savannah Gonzales, Vanessa Brizuela, Steve Daly, Yoon Jae Chang, Mahdote Abebe, Yash Rajana, Kelly Wichmann, Catheryn Bolick, Jaiyana King, Mark Fiala, Julie Fortier, Reyka Jayasinghe, Mark Schroeder, Li Ding, Ravi Vij, Jessica Silva-Fisher

**Affiliations:** 1 Department of Internal Medicine, Division of Oncology, School of Medicine, Washington University in St. Louis, MO, 631101; 2 Siteman Cancer Center, Washington University in St. Louis, MO, 631102

## Abstract

Multiple Myeloma (MM) is an incurable form of cancer that arises from malignant plasma cells, with over 35,000 new cases diagnosed annually in the United States. While there are a growing number of approved therapies, MM remains incurable and nearly all patients will relapse and exhaust available treatments. Mechanisms for disease progression are unclear and little is known regarding the role of long non-coding RNAs (lncRNA) in mediating disease progression and response to treatment. Here, we used transcriptome sequencing to compare newly diagnosed MM (NDMM) patients who had short progression-free survival (PFS) to standard first-line treatment (PFS < 24 months) to patients who had prolonged PFS (PFS > 24 months). We identified 157 differentially upregulated lncRNAs with short PFS and focused our efforts on characterizing the most upregulated lncRNA, *LINC01432*. We investigated *LINC01432* to show that its overexpression significantly increases cell viability and reduces apoptosis, while knockdown significantly reduces viability and increases apoptosis. Next, we show that *LINC01432* directly interacts with the RNA binding protein, CELF2. Lastly, we showed that *LINC01432*-targeted locked nucleic acid antisense oligonucleotides reduce viability and increases apoptosis. In summary, this fundamental study identified lncRNAs associated with short PFS to standard NDMM treatment and further characterized *LINC01432*.

## Introduction

Multiple myeloma (MM) is a prevalent disease and is the fifteenth leading cause of cancer-related deaths in the United States.(1) Despite the increasing availability of treatment regimens, nearly all patients with MM become refractory and die from the disease or its sequel.(2–4) In addition, while some improvements in patient outcomes have been achieved using novel immunomodulatory agents, new approaches are still needed due to high toxicity and the development of drug resistance. (5–7) This underscores the need for new therapeutic approaches. Thus, knowledge surrounding the mechanisms and biomarkers of treatment resistance in MM patients are critically needed to support the development of novel MM therapies.

Long non-coding RNA (lncRNA) is defined as RNA greater than 200 nucleotides in length that is not translated into functional proteins. Prior studies have reported that lncRNAs can promote the pathogenesis of all cancer types including MM.(8–12) Many lncRNAs have also been shown to promote MM drug resistance, including *NEAT1, ANRIL, MEG3, LINC00461, H19*, and *PCAT1*.(8, 13–17) The subcellular localization of a lncRNA is highly important and specific to its biological functions in the cell, which may include transcriptional regulation, translational regulation, and interaction with RNA binding proteins.(18, 19) Further, recent advances in understanding the functions and crucial roles lncRNAs play in promoting cancer, including MM, increases their potential as targets for RNA-based therapeutics.(20–22)

In this study, we used RNA sequencing data from a cohort of NDMM patients to identify lncRNAs that were associated with a short progression-free survival (PFS). We identified several lncRNAs that were highly upregulated in patients with short PFS, as compared to prolonged PFS, and determined that *LINC01432* bound to the RNA-binding protein, CELF2, to inhibit apoptosis and increase viability.

## Methods

### RNA sequencing data, patient samples, and cell lines.

RNA sequencing data from NDMM patients were obtained from the Multiple Myeloma Research Foundation (MMRF) Clinical Outcomes in Multiple Myeloma to Personal Assessment of Genetic Profiles (CoMMpass) study (https://registry.opendata.aws/mmrf-commpass), accessed in February of 2021 (**Supplementary Figure 1, Supplementary Tables 1 and 2**). MM cell lines were generously provided by Dr. John DiPersio at Washington University in St. Louis (RPMI 8226, U266B1, MM1.S, and OPM2) and were all cultured in RPMI 1640 media (Invitrogen, Carlsbad, CA) supplemented with 15% fetal bovine serum (Invitrogen) and 1% penicillin/streptomycin (Invitrogen). MM1.R cell lines were purchased from ATCC (catalog number CRL-2975). NDMM patient bone marrow aspirates were obtained from the Multiple Myeloma Tissue Banking Protocol (IRB 201102270) processed by the Siteman Cancer Center Tissue Procurement Core.

Full length *LINC01432* transcript was amplified via PCR and cloned into the pCFG5-IEGZ-GFP vector (generously provided by Dr. Chris Maher, Washington University in St. Louis, Piscataway, NJ) to create the pCFG5-IEGZ-GFP-Luc-*LINC01432* vector (pCFG5-*LINC01432*), as previously described(23). Full vector length was confirmed by GeneScript. Validated cell lines showing high levels of *LINC01432* expression by RT-qPCR, as compared to empty vector, were used for subsequent assays.

*LINC01432* knockdown CRISPR/Cas9 cells were generated using the RPMI 8226 cell line. The sgRNAs were generated by the Genome Engineering and Stem Cell Center, Washington University in St. Louis. sgRNAs were cloned into the pLV hUbC-dCas9 KRAB-T2A-GFP plasmid (Addgene #672620). HEK 293T cells were infected with this lentivirus to induce expression of dCas9-KRAB,(24) followed by transduction, similar as above into RPMI 8226 cells and validated knockdown of *LINC01432* expression via RT-qPCR.

### Transfection of locked nucleic acid antisense oligonucleotides.

Locked nucleic acid GapmeR antisense oligonucleotides (LNA ASOs) targeting *LINC01432* (Qiagen, cat# 3653410) and CELF2 (Qiagen, cat# 339511), and negative control LNA ASOs (Qiagen, cat#148759394), were designed using the Qiagen Antisense LNA GapmeR Custom Builder (https://www.qiagen.com), sequences are listed in **Supplementary Table 3**. MM cells were seeded at a density of 500 000 cells/well in 6-well plates, transfected with respective ASOs at 50nM-100nM concentration using Lipofectamine 2000, and incubated for 48–72 hours. Cells were harvested and target knockdown was validated via RT-qPCR.

### RNA Sequencing data analyses.

RNA sequencing data was processed and analyzed from the MMRF ComPASS study. Briefly, the GRCh37 reference genome (hs37d5 version)(25) was used for assembly, Ensemble version 74 was used, along with additional annotations provided in the GTF file (https://github.com/tgen/MMRF_CoMMpass.git). To ensure the rigor of these data, we performed quality control checks on the RNA BAM files using Picard RNA metrics and BamTools Ig Counts. DAVID(26) was used to determine Gene Ontology and pathway analyses. POSTAR3(27) was used to determine *LINC01432* RNA:protein binding.

### Multiplexed Fluorescent RNA in situ Hybridization (mFISH).

RNAScope was performed as previously described,(28) with some modifications using RNAscope 2.5 HD Reagent Kit Red assay combined with Immunohistochemistry (Advanced Cell Diagnostics [ACD], Catalog #323180 and #322372) according to manufacturer’s instructions. Briefly, bone marrow aspirates or isolated tumors were applied to slides, baked, deparaffinized, hydrogen peroxide was applied, and co-detection target retrieval was performed. Slides were then incubated overnight with CUGBP2 (CELF2) antibody (Protein Tech, Cat#12921–1-AP) in a HybEz Slide Rack and incubated overnight at 4°C. The next day, slides were washed then post-primary fixation was performed. Probes for *LINC01432* (ACD Cat# 878271) were then hybridized and RNA was then serial amplified and stained with Fast Red solution. Slides were blocked and secondary Alexa Fluor 488 antibody (Abcam, cat#ab150081) was applied for one hour at room temperature in the dark. Finally, slides are counter stained with DAPI (Sigma, cat#D9542) and mounted with ProLong Gold Antifade Reagent (Invitrogen, cat#P36930). Slides were imaged on the EVOS M5000 Imaging System (Invitrogen).

Analysis of mFISH and IHC images was performed by comparing expression of *LINC01432* or CELF2 between different cell lines or tissues and simultaneously verifying their cellular localization or intensity of expression. We first visualized our target RNA molecules using an EVOS M5000 imaging system and quantified targets with QuPath Software v0.5.1 to obtain cell count per region and number of spots per cell data. We then applied multiplex analysis followed by the cell distribution analysis for detecting lncRNA spots in each cellular compartment.

### In vitro phenotypic assays.

We used the ApoTox-Glo Triplex Assay (Promega, Madison, WI) to simultaneously measure viability, cytotoxicity, and apoptosis in the same sample. We seeded 20 000 cells/well of Control CRISPR/Cas9, *LINC01432* knockdown CRISPR/Cas9, empty vector, *LINC01432* overexpression, or wild-type RPMI 8226 cells in triplicate into a 96-well plate at 100uls complete media per well. We began by first adding 20ul of Viability/Cytoxicity reagent to all wells, and briefly mixing for ~30 seconds. Plates were then placed in a 37°C incubator for one hour. Next, we measured the intensity of fluorescence (relative fluorescence units) using 400_Ex_/505_Em_ (viability) and 485_Ex_/520_Em_ (Cytotoxicity) in Varioskan LUX microplate reader. To measure apoptosis, we next add 100ul Caspase-Glo 3/7 reagent to the same wells and briefly mixed by orbital shaking ~20 seconds, followed by incubation at room temp for 30 minutes. Luminescence (relative luminescence units) was then measured using Varioskan LUX microplate reader to detect caspase activation.

We measured apoptosis by isolating cells and assessing via flow cytometry using BD Horizon V450 AnnexinV (BD Biosciences, Franklin Lakes, NJ). We seeded 500 000 cells/well in a 6-well plate for 24 hours. Cells were then harvested and the usual protocol was followed, per manufactures instructions. Briefly, cells were washed twice with PBS, then incubated V450 AnnexinV and Propidium Iodide (ThermoFisher) for fifteen minutes at room temperature in the dark. Apoptosis and DNA content was assessed on a flow cytometer machine (Novios, Becton Dickinson) by the Flow Cytometry Core of Siteman Cancer Center, Washington University in St. Louis. We collected a minimum of 50 000 cells per sample in triplicate. FlowJo Version 10 (Becton Dickinson) was used to analyze data.

### In vivo individual-nucleotide resolution cross-linking immunoprecipitation (iCLIP).

The iCLIP assay was performed as previously described.(23) Briefly, cells were washed with cold PBS and irradiated with 150 mJ/cm^2^ of UVA (254 nm) in a crosslinker device (Stratalinker). Cell pellets were resuspended in 1ml of NP-40 lysis buffer (20mM Tris–HCl at pH 7.5, 100mM KCl, 5mM MgCl2, and 0.5% NP-40) with 1μl protease inhibitor and 1mM DTT, incubated on ice for ten minutes, and then centrifuged. Supernatants were collected, 1U/μl RNase T1 was added, then cell lysates were incubated at 22°C for 30 minutes. Protein G Beads were resuspended in 100μls NT2 buffer (50mM Tris–HCl at pH 7.5, 150mM NaCl,1mM MgCl2, 0.05% NP-40) with 5μg of respective antibodies, then rotated for one hour at room temperature. All antibodies are listed in **Supplementary Table 4**. Cell lysates were added to the beads and incubated for three hours, the beads were washed with NT2 buffer, and then incubated with 20 units RNAse-free DNase I for 15 minutes at 37°C in a thermomixer, shaking slowly. Protein kinase buffer (141μls NP-40 lysis buffer, 0.1% SDS, 0.5 mg/ml Proteinase K) was then added. Supernatants were then collected and RNA isolation was performed using a standard phenol:cholorform:isoamyl alcohol protocol. RNA was then reverse transcribed using SuperScript III First strand cDNA system, as per manufacturer’s protocol (ThermoFisher) and primers tiling *LINC01432* (**Supplemental Table 3**) were used to detect *LINC01432*:protein binding.

### In vivo myeloma models.

All animal experiment protocols in this study were reviewed and approved by the Institutional Animal Care and Use Committee of Washington University in St. Louis. For subcutaneous injections, 2e^5^–1e^7^ cells (RPMI 8226 wild-type, U266B1 wild-type, Control CRISPR/Cas9, *LINC01432* knockdown CRISPR/Cas9, empty vector, or *LINC01432* overexpression) were subcutaneously injected NOD/SCID/γc^−/−^ (NSG) mice (N = 5–10 per group). Resulting tumor size was quantified weekly via caliper measurements, comparing length x width x height x 0.5. For post-analyses, subcutaneous tumor tissues were removed after sacrifice, formalin fixed, and paraffin embedded. This experiment was repeated twice.

### Data sharing statement

All RNA sequencing data is available at GEO under accession number GSE267013. All other data supporting the findings of this study are available within this article and its Supplementary Information files from the corresponding author, upon reasonable request.

## Results

### Identification of dysregulated lncRNAs with short progression-free survival to standard MM therapy.

**Figure 1:**
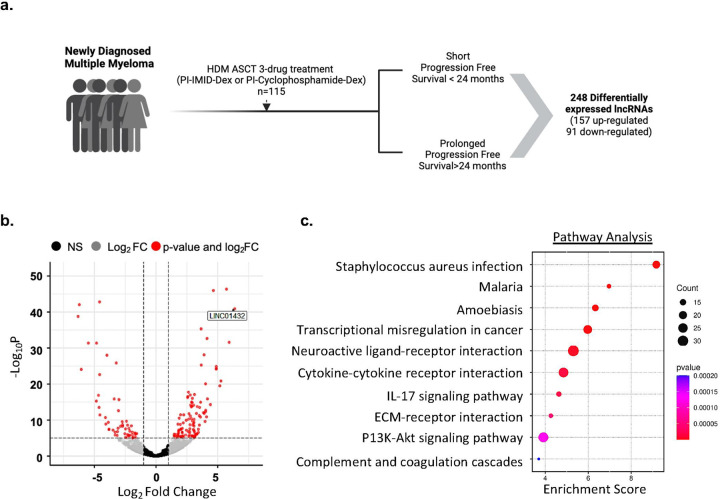
Identification of dysregulated lncRNAs associated with short progression-free survival to MM therapy

In order to identify lncRNAs that are differentially expressed in patients that exhibit a short progression-free survival (PFS) to the standard MM treatment approach, we analyzed transcriptome sequencing data from CD138+ bone marrow samples obtained from 115 NDMM patients in the MMRF CoMMpass study (**Supplementary Figure 1)**. We assigned samples to one of two groups based on each patient’s length of PFS to standard MM therapy, short, those who had progression-free survival < 24 months (short PFS) from the first dose of MM treatment (N = 38), and prolonged progression-free survival, those with > 24 months PFS (N = 77), Table 1, **Supplemental Table 1**. We identified 157 upregulated and 91 downregulated lncRNAs in short PFS, as compared to prolonged PFS (log2Fold Change > +/−2, *p* < 0.05), Figure 1a. lncRNAs identified as being most differentially expressed in short PFS included *LINC01432, lnc-LGALS9B-7, LINC01916, Lnc-SPIDR-1, and MAGEA4-AS1*, Figure 1b. We also identified two lncRNAs previously reported to be associated with MM, MEG3(16, 29), and H19,(30, 31) **Supplemental Table 1.** Next, we performed pathway analysis on all differentially expressed RNAs in short PFS to identify highly enriched gene sets associated with, but not limited to, staphylococcus aureus infection (*p* = 6.75e^−10^), transcriptional dysregulation in cancer (*p* = 1.05e^−06^), cytokine-cytokine receptor interactions (*p* = 1.42e^−05^), IL-17 signaling pathway (*p =* 2.31e^−05^), and ECM-receptor interactions (*p =* 5.5e^−05^), Figure 1c. Gene ontology analysis further showed high enrichment of more than one pathway associated with immune response, B cell mediated immunity, and multiple hemoglobin complexes, **Supplementary Figure 2, a and b**. This analysis of sequencing data from NDMM patients in the MMRF CoMMPass study allowed us to identify lncRNAs that are differentially expressed in patients that exhibit a short PFS to standard MM treatment.

### LINC001432 is the top most significantly upregulated lncRNA in patients with short PFS.

**Figure 2:**
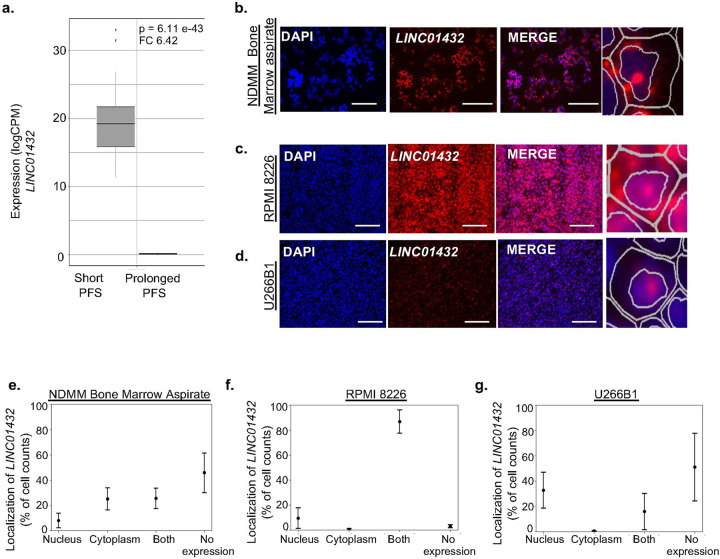
*LINC01432* is the most upregulated lncRNA in patients with short progression-free survival to MM therapy

We focused our subsequent experimental analyses on characterizing the most significantly upregulated lncRNA in short PFS, as compared to prolonged PFS, termed *LINC01432 (*Fold change = 6.42, *p* = 6.11e^−43^), Figure 1b and Figure 2a. *LINC01432* is a long intergenic non-protein coding RNA located on chromosome 20, has four exons, and is 693 nucleotides long. There is little-to-no current knowledge about *LINC01432*; it has only been reported to contain a SNP associated with male baldness in a single-trait genome-wide association study.(32) Due to the heterogeneity and hyperploidy in several chromosomes observed in MM patients, we began by characterizing *LINC01432* by assessing different genetic subtypes of MM. We found that high expression of *LINC01432* was correlated with t(14;16) and Amp (1q) translocations (t[14;16] positive correlation = 0.57; Amp [1q] positive correlation = 0.14), **Supplementary Figure 3a**.

To further characterize *LINC01432*, we analyzed its expression in a panel of MM cell lines and found that *LINC01432* is highly expressed in RPMI 8226 and OPM-2 cells, with low level expression detected in MM.1S, MM.1R, and U266B1 cells, **Supplementary Figure 3b**. Next, we confirmed the expression of *LINC01432* in NDMM bone marrow aspirates using mFISH, Figure 2, b and e. To assess the clinical significance of *LINC01432* in the context of MM, we subcutaneously injected mice with the MM cell lines RPMI 8226 and U266B1 to assess *in vivo* tumor growth and *LINC01432* expression. We found high expression of *LINC01432* in RPMI 8226 tumors and low expression in U266B1 tumors using mFISH, Figure 2, c and d. We determined that *LINC01432* is localized in both the cytoplasm and the nuclear compartments of RPMI 8226 cell line tumors, with 9.50% of cells exhibiting expression in the nucleus, 0.59% exhibiting expression in the cytoplasm, 86.91% exhibiting expression in both compartments, and 2.99% with no apparent *LINC01432* expression, Figure 2f. In the U266B1 cell line, which has low endogenous *LINC01432* expression levels, *LINC01432* expression was located in the nucleus in 32.79% of cells, in the cytoplasm of 0.44% of cells, in both compartments of 15.83% of cells, and expression was not detected in 50.94% of cells, Figure 2g. These data indicate that *LINC01432* is highly expressed in NDMM patient samples and in MM cell lines and is a novel lncRNA expressed in patients with short PFS to standard treatment.

### LINC01432 inhibits apoptosis and increases tumor growth.

**Figure 3:**
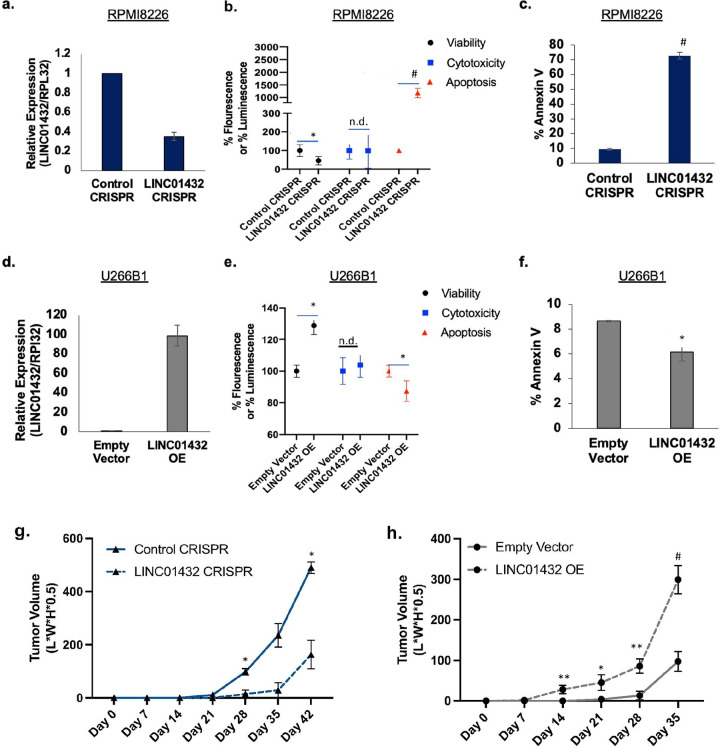
*LINC01432* promotes an aggressive phenotype

To investigate the molecular mechanisms through which *LINC01432* may induce a short PFS to standard MM therapy and to test its potential as a therapeutic target, we used CRISPR/Cas9 (CRISPR) to knockdown *LINC01432* expression in RPMI 8226 cells, Figure 3a, that have high endogenous expression levels, **Supplementary Figure 3b**. The development of a knockout cell line was unsuccessful due to a result of high cell death. Using the ApoTox-Glo Triplex Assay, we found that *LINC01432* knockdown significantly decreased viability (*p* = 0.03) and significantly increased apoptosis (*p* = 2.69e^−05^) in these cells, as compared to control CRISPR cells, Figure 3b. Increased apoptosis in *LINC01432* knockdown cells was further validated via Annexin V flow cytometry (*p* = 1.25e^−06^), Figure 3c. We then generated *LINC01432* overexpression from the U266B1 cell line, which have low endogenous *LINC01432* expression levels (Figure 3d and **Supplementary Figure 3b**) and found that these cells have significantly increased viability (*p* = 0.001) and significantly decreased apoptosis (ApoTox-Glo *p* = 0.04, AnnexinV *p* = 0.04), as compared to empty vector control cells, Figure 3, e and f. Next, we subcutaneously injected mice with *LINC01432* knockdown and control CRISPR cell lines and compared *in vivo* tumor growth **Supplementary Figure 3c**. This revealed significantly lower tumor volume in mice injected with *LINC01432* knockdown cells, as compared to control cells (Day 28 *p* = 0.04, Day 42 *p* = 0.02), Figure 3g and **Supplementary Figure 3d**. We similarly injected mice with *LINC01432* overexpression cells and empty vector controls and found that overexpression resulted in significantly higher tumor volume, as compared to controls (Day 14 *p* = 0.009, Day 21 *p* = 0.04, Day 28 *p* = 0.003, Day 35 *p* = 0.000), Figure 3h and **Supplementary Figure 3e**.

Next, we assessed the effects of *LINC01432* knockdown and overexpression on apoptotic markers, including *TP53* pathway genes, via RT-qPCR. We found that expression of apoptotic markers was significantly higher in RPMI 8226 *LINC01432* knockdown cells, as compared to controls (*TP53 p* = 0.004, *cMYC p* = 0.0002, *BAX p* = 5.96e^−13^). Similarly, we found that expression of apoptotic markers was significantly lower in tumors arising from *LINC01432* overexpression cells, as compared to controls (*TP53 p* = 2.04e^−09^, *cMYC p* = 1.57e^−10^, *BAX p* = 1.07e^−07^), **Supplementary Figure 4, a and b**. In addition, we detected a decrease in *γH2AX*, a marker of DNA double-stranded breaks, in tumors arising from *LINC01432* overexpression cells, **Supplementary Figure 4c**. These data provide evidence that *LINC01432* is highly expressed in MM cell lines and its expression is associated with increased viability and decreased apoptosis.

### LINC01432 binds to CELF2 protein.

**Figure 4:**
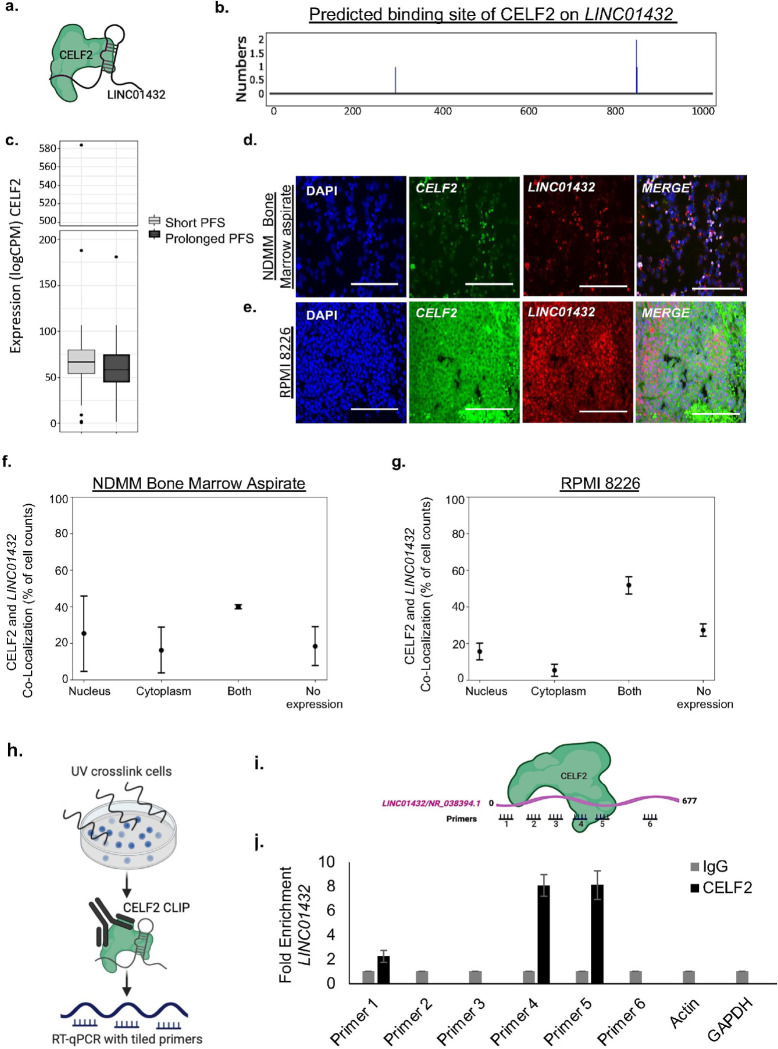
CELF2 binds to *LINC01432*

Many functional studies of lncRNAs, including our group’s research, have found that the ability of lncRNAs to bind with proteins and regulate downstream genes is integral to their roles in cancer and therapeutic resistance.(23, 33) As limited functional data on *LINC01432* is available, we utilized POSTAR3(27) as a first step to identifying proteins which potentially bind to *LINC01432*. This analysis identified that *LINC0143*2 to bind to the CELF2 RNA-binding protein from a publicly available CLIP-sequencing dataset (34), **Supplementary Figure 5**, Figure 4, a and b. Although the determined binding score was low (0.019), CELF2 (CUGBP Elav-like family) proteins are RNA-binding proteins with pleiotropic capabilities in RNA processing that have been found to compete with non-coding RNAs, including lncRNAs.(35) CELF2 has been shown to bind lncRNAs to regulate downstream mRNAs, thereby promoting proliferation, migration, and tumor growth of multiple cancers,(36–40) however, this has not yet been studied in MM. Thus, we investigated whether *LINC01432* binds to CELF2.

Analysis of our NDMM patient RNA sequencing dataset revealed high level expression of CELF2 (logCPM > 50), but no significant differences in expression levels were identified between short PFS and prolonged PFS, Figure 4c. The Human Protein Atlas (proteinatlas.org) indicates that CELF2 expression is enriched in bone marrow (Tau score = 0.40) and localized to the nucleoplasm, vesicle, and midbody ring. Assessment of CELF2 expression in multiple blood cancer types indicated that CELF2 is highly expressed in leukemia, lymphoma, and MM (logCPM >10), **Supplementary Figure 6a**. Western blot analysis of CELF2 protein expression in whole cell lysates showed slight increased expression of CELF2 in both *LINC01432* RPMI 8226 knockdown (Fold Change = 1.41) and U266B1 overexpression cells (Fold Change = 1.58), as compared to controls, **Supplementary Figure 6, b and c.** mFISH analysis using *LINC01432* probes in combination with CELF2 protein immunohistochemistry in NDMM bone marrow aspirates indicated that CELF2 is expressed in both the nucleus and cytoplasm, Figure 4, d and f. Further, we found evidence of CELF2 and *LINC01432* co-localization in both cellular compartments in RPMI 8226 wild-type Figure 4, e and g, and U266B1 *LINC01432* overexpression cells, **Supplementary Figure 7**.

Next, we conducted iCLIP analysis to identify the regions of *LINC01432* that may be directly bound by CELF2, which combines UV cross-linking with immunoprecipitation and RT-qPCR to precisely map the binding sites of RNA-binding proteins, Figure 4h. RT-qPCR tiling primers spanning *LINC01432* showed direct binding of CELF2 to Tiling Primer 1 (Fold Change > 2), Tiling Primer 4 (Fold Change > 8), and Tiling Primer 5 (Fold Change > 8), as compared to IgG negative control, in RPMI 8226 cells with high level endogenous expression of *LINC01432*, Figure 4, i and j. These data indicate that *LINC01432* is bound by CELF2 protein in MM cell lines.

### Treating cells with LINC01432 locked nucleic acid antisense oligos (LNA ASOs) increases apoptosis.

**Figure 5:**
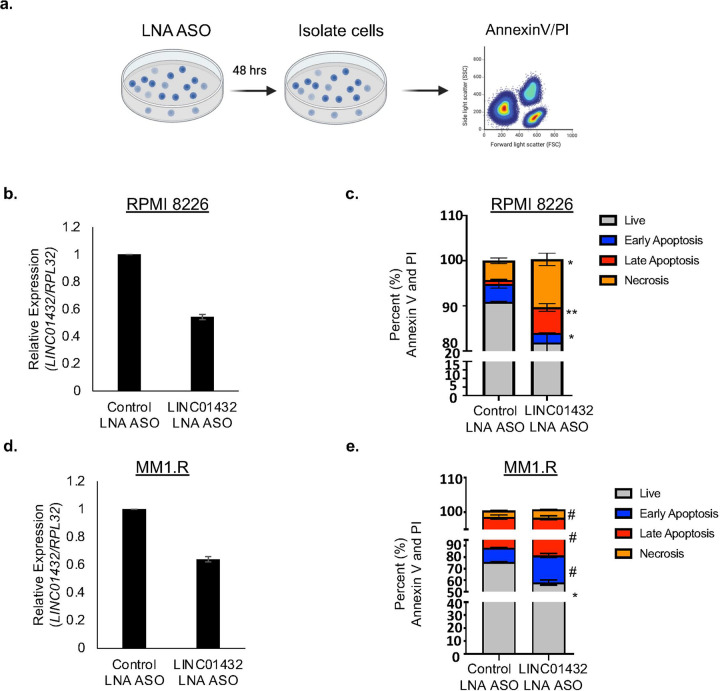
LNA ASO-mediated *LINC01432* knockdown induces apoptosis

To assess the potential of *LINC01432* as a therapeutic target, we developed *LINC01432*-targeted LNA ASOs along with control LNA ASOs.(41, 42) We treated RPMI 8226 cells and MM1.R cells with these LNA ASOs, Figure 5a, and confirmed knockdown of *LINC01432* expression, Figure 5, b and d. We next showed that LNA ASO-mediated *LINC01432* knockdown increased the proportion of cells in early apoptosis (MM1.R *p* = 0.003), late apoptosis (RPMI 8226 *p* = 0.005, MM1.R *p* = 0.005), and necrosis apoptosis (RPMI 8226 *p* = 0.013, MM1.R *p* = 0.02), as measured by flow cytometry, Figure 5, c and d. Further, we showed that LNA ASO-mediated CELF2 knockdown increased apoptosis in MM cell lines, **Supplementary Figure 8, a and b**. These data provide evidence that *LINC01432* inhibits apoptosis.

**Figure 6:**
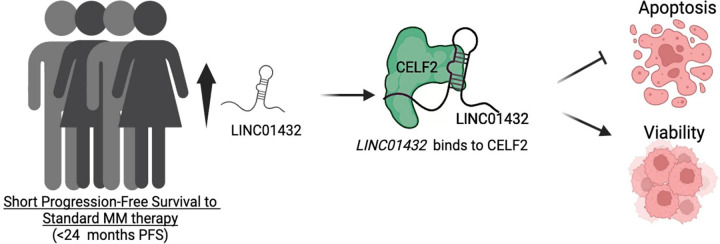
Schematic of overall outcome

In summary, our study identified differentially expressed lncRNAs associated with patients who had short PFS to standard MM therapy and determined that *LINC01432* bound to CELF2 and that LNA knockdown in MM cells promotes apoptosis and decreases viability suggesting the role of *LINC01432* and the *LINC01432*-CELF2 complex in promoting PFS to the standard treatment, Figure 6.

## Discussion

While some advances in MM treatment are emerging through clinical trials of novel cellular immunotherapies targeting immune cells (43–45)unfortunately, interpatient heterogeneity has hindered the elucidation of the molecular mechanisms that control the progression of plasma cells in patients with MM. Thus, knowledge surrounding mechanisms and biomarkers related to treatment resistance in MM patients are critical for novel therapy development.

In this study, we identified differentially expressed lncRNAs in NDMM patients who exhibited a short PFS to the standard MM therapy. The most upregulated annotated lncRNA, *LINC01432*, was found to bind to the CELF2 protein, leading to inhibition of apoptosis and promotion of cell viability. CELF2 has been previously reported to bind to lncRNAs and regulate downstream mRNAs in multiple forms of cancer,(39, 40, 46, 47) however, this has not yet been studied in the context of NDMM. In cancer, CELF2 is found to be localized to the nucleus, where it is associated with alternative splicing and transcript editing, in RNA granules, where it regulates mRNA stability, and in the cytoplasm, where it regulates pre-miRNA maturation, translation, and alternative polyadenylation.(34, 48, 49) Here, we determined that CELF2 shows different patterns of expression in MM cell lines with differential levels of endogenous *LINC01432* expression. In the presence of *LINC01432,* CELF2 was localized to the cytoplasm. We observed that co-localization allowed the binding of *LINC01432* to CELF2 to inhibit apoptosis, although more studies need to be conducted to determine if this is a result of the specific interaction or is solely dependent on the increased expression of *LINC01432*. In addition, POSTAR3 also predicted *LINC01432* binding to the protein AGO2, thereby we believe that there may be additional proteins that may bind to *LINC01432,* as lncRNAs are known to interact with multiple RNA binding proteins. As we are in the earliest stages of understanding *LINC01432* tumor biology, this study allows us to predict that *LINC01432-*CELF2 interaction may play a larger role in the pathogenesis of MM. Future studies are needed to better understand this interaction and other potential protein interactions in MM and to fully characterize its role in the development of resistance to chemotherapy.

One promising aspect of lncRNAs that makes them ideal novel targets for the development of RNA therapeutics is their tissue and cell specific expression patterns (50, 51). ASOs have been shown to be a powerful tool for therapeutically targeting lncRNAs.(52, 53) This study represents a preliminary investigation into the use of LNA ASO to downregulate *LINC01432* lncRNA. Future studies are being conducted to provide evidence of its clinical significance. Many unanswered questions remain regarding the exact mechanism through which *LINC01432* regulates downstream pathways and mediates epigenetic regulation while bound to CELF2. In conclusion, our study provides preliminary insights into the role of lncRNA expression in NDMM patients who exhibit a short PFS to standard MM therapy and identifies a novel potential target for the development of future MM therapies.
